# Orthorexic Tendencies Are Associated with Autistic Traits in Patients with Borderline Personality Disorder

**DOI:** 10.3390/jcm14113891

**Published:** 2025-06-01

**Authors:** Liliana Dell’Osso, Benedetta Nardi, Federico Giovannoni, Chiara Bonelli, Gabriele Massimetti, Ivan Mirko Cremone, Stefano Pini, Barbara Carpita

**Affiliations:** Department of Clinical and Experimental Medicine, University of Pisa, 56126 Pisa, Italy; liliana.dellosso@unipi.it (L.D.); f.giovannoni10@gmail.com (F.G.); chiarabonelli.95@hotmail.it (C.B.); gabriele.massimetti@unipi.it (G.M.); ivan.cremone@gmail.com (I.M.C.); stefano.pini@unipi.it (S.P.); barbara.carpita@unipi.it (B.C.)

**Keywords:** orthorexia nervosa, orthorexia, borderline personality disorder, autistic traits, autism spectrum, eating disorders

## Abstract

**Background/Objectives**: Orthorexia Nervosa (ON), a condition marked by an obsessive focus on eating healthily, has drawn increasing clinical attention due to its rigid dietary patterns and social impairment. Borderline Personality Disorder (BPD), characterized by emotional dysregulation, impulsivity, and unstable interpersonal relationships, frequently co-occurs with eating disorders. Recent research suggests that autistic traits—such as cognitive rigidity and restricted interests—may underlie both ON and BPD, especially in female populations. This study aimed to assess the prevalence of orthorexic tendencies in patients with BPD compared to healthy controls (HCs) and to explore their associations with autistic traits and disordered eating behaviors. **Methods**: This study involved 73 BPD patients and 52 HCs. Participants completed the Adult Autism Subthreshold (AdAS) Spectrum, Eating Disorder Inventory-2 (EDI-2), and the ORTO-15 questionnaire. **Results**: BPD patients scored significantly higher than HCs on AdAS Spectrum and EDI-2, and significantly lower on ORTO-15, indicating more pronounced autistic traits, disordered eating behavior, and orthorexic tendencies. A greater proportion of BPD individuals reported clinically relevant ON symptoms according to the ORTO-15 threshold. Orthorexic symptoms were significantly correlated with most EDI-2 and all AdAS Spectrum domains. Regression analysis revealed that autistic traits, but not feeding and eating disorder symptoms, significantly predicted orthorexic tendencies. **Conclusions**: Orthorexic tendencies are more prevalent in individuals with BPD and are significantly associated with autistic traits. These findings suggest that ON may represent a manifestation of the autism spectrum, particularly in individuals with BPD, and support a reconceptualization of ON within a neurodevelopmental framework. **Recommendation**: Future research is needed in order to clarify the temporal and causal relationships among autistic traits, BPD symptomatology, and the emergence of orthorexic behaviors.

## 1. Introduction

The Diagnostic and Statistical Manual of Mental Disorders (DSM) classifies Borderline Personality Disorder (BPD) as a personality disorder characterized by a persistent pattern of instability in interpersonal relationships, self-image, and mood, typically beginning in early adulthood [1]. Core features of individuals with BPD include marked impulsivity, intense feelings of anger and emptiness, fear of abandonment, emotional dysregulation, self-harming behaviors or suicidal threats, and occasional episodes of paranoia or dissociation. The lifetime prevalence of BPD is estimated at 5.3%, with women representing about 80% of those affected [2]. Personality disorders, including BPD, are common in clinical settings, with approximately 45% of outpatient cases involving personality disorders alongside other co-occurring conditions [3]. Due to its early onset, high prevalence, and strong association with suicide risk, BPD has garnered significant research attention and poses a major public health concern. It requires specialized treatment and continuous monitoring. Moreover, BPD is frequently associated with other psychiatric conditions, including anxiety disorders, mood disorders, additional personality disorders, substance use disorders, and eating disorders. Indeed, some studies suggest that comorbidity rates in BPD can exceed 70%, highlighting that co-occurring mental health issues are the norm rather than the exception [2,4].

In particular, recent scientific research has increasingly highlighted a strong connection between BPD and Feeding and Eating Disorders (FED), which are characterized by disordered eating behaviors that significantly impair physical health or psychosocial functioning [1,5,6]. Indeed, many studies have indicated that individuals with BPD often exhibit disordered eating behaviors, regardless of whether they have a formal diagnosis of an eating disorder [7]. These behaviors include binge eating, frequent dieting, meal skipping, and self-induced vomiting, among others. BPD is particularly linked to impulsive eating patterns such as binging and purging [8]. A recent review of empirical studies examining personality disorders in relation to eating disorders in adults found that BPD was the most common co-occurring personality disorder among those with pathological eating behaviors [9]. Although the causes of BPD and FED have only been partially understood, negative childhood experiences—such as bullying, neglect, and various forms of abuse (emotional, physical, and sexual)—appear to be crucial in the development of both disorders [10,11]. Trauma, in particular, seems to contribute to emotional dysregulation, leading to outbursts of anger and self-harming behaviors, episodes of dissociation and depersonalization, and chronic feelings of guilt, despair, and shame. These factors are often accompanied by trust and intimacy issues, somatization, as well as negative expectations about life—all of which are common in individuals with both FED and BPD [5,12,13].

In this framework, some recent studies suggest that the correlation between FED and BPD may be mediated by the presence of autistic traits [14,15,16,17,18]. Specifically, recent research has proposed that certain cases of BPD and eating disorders could be reconceptualized as a typically female presentation of the autism spectrum. In the case of BPD, numerous studies report a significant overlap in symptoms, such as intense, unstable relationships, superficial friendships, high rates of self-injurious behaviors, difficulties in both verbal and non-verbal communication, and elevated levels of autistic traits among individuals with BPD [19,20,21]. Similarly, individuals with FEDs exhibit core characteristics of the autism spectrum, including cognitive rigidity, impaired theory of mind, and intense, narrow interests that often focus on food, while, on the other hand, rigid behaviors and selectivity about food are often reported in individuals on the autism spectrum [15,22,23,24]. Given these overlaps, it has been proposed that these disorders may represent expressions of the female autistic phenotype. This phenotype is characterized by a stronger drive for social connection, and a greater use of social camouflaging strategies in order to cope with social difficulties, together with a different pattern of restrictive interests with respect to those reported in autistic males, including an increased focus of food and diet, and a higher likelihood of internalizing disorders, which increases the risk to pass under noticed and of developing anxiety, mood disorders, self-destructive behaviors, and eating disorders [15,25,26].

In this framework, a possible emerging manifestation of eating disorders, Orthorexia Nervosa (ON), has recently gained attention in both clinical and public spheres. First introduced by Bratman in 1997, ON is defined by an obsessive focus on the purity of food, with individuals prioritizing the quality of what they eat over taste [27]. This intense preoccupation with food selection, preparation, and rituals can lead to a rigid and restrictive eating pattern that disrupts daily life. People with ON often avoid social situations like dining out, as their strict eating habits interfere with social interactions [27,28]. Moreover, those with ON may view their self-imposed, “healthier” dietary choices as morally superior, which can foster a sense of separation from others and create a dichotomous worldview [29,30]. While research indicates that the prevalence of ON is rising, estimates vary widely, ranging from 2.6% to 87.7% [31,32]. However, to date, there is currently no consensus on its formal diagnostic criteria.

Although research on ON is still limited, studies are suggesting a potential correlation between ON and BPD, similar to other FEDs [33]. Increasing evidence also points to a connection between ON and autistic traits, highlighting an association between ON symptoms and autistic traits in different samples [29,30,34,35]. From a symptomatological viewpoint, the intense focus on healthy eating, ritualized behaviors related to food preparation and consumption, and a selective, restricted interest in diet exhibit may overlap with some autism spectrum features. Additionally, the sense of moral stiffness and intolerance toward others’ eating habits often seen in ON could be linked to social and emotional reciprocity deficits typically found in individuals with autism spectrum [29,30,34,35].

Given these premises, the aim of this study was to assess the presence of orthorexic tendencies in individuals with BPD compared to healthy controls (HC), focusing also on evaluating the association between orthorexic tendencies, autistic traits, and other eating disorders symptomatology in this sample.

## 2. Materials and Methods

### 2.1. Study Sample and Procedures

A total of 52 HCs and 73 consecutive BPD patients undergoing treatment at the University of Pisa Psychiatric Clinic were chosen for this study. The participants were between the ages of 18 and 65. The study excluded participants who were younger than 18 years old, had a history of substance abuse, schizophrenia, severe neurological or medical conditions, significant intellectual or language impairments, or were incapable of completing psychometric questionnaires or giving written consent on their own.

Before completing the informed consent form, participants received thorough information about the study and had a chance to ask questions.

The study was approved by the local ethical committee and complied with the Declaration of Helsinki’s ethical guidelines.

### 2.2. Measures

#### 2.2.1. Adult Autism Subthreshold (AdAS) Spectrum

The AdAS Spectrum is a self-report tool, developed in order to detect full- and subthreshold autistic symptoms and characteristics in individuals without intellectual disability. The questionnaire is composed of 160 dichotomous items organized into seven domains: Childhood/Adolescence, Verbal and Non-verbal Communication, Empathy, Inflexibility and Adherence to Routine, Restricted Interests and Rumination, and Hyper–Hypo Reactivity to Sensory Input. During the validation study, the questionnaire exhibited good internal consistency (Kuder–Richardson’s coefficient = 0.964), great test–retest reliability (ICC = 0.976), and strong convergent validity with existing dimensional measures of autism [36].

#### 2.2.2. The Eating Disorder Inventory (EDI-2)

The EDI-2 is a self-administered questionnaire containing 91 items, which are categorized into 11 different areas: Drive for Thinness, Bulimia, Body Dissatisfaction, Ineffectiveness, Perfectionism, Interpersonal Distrust, Interoceptive Awareness, Maturity Fears, Asceticism, and Impulsivity. Respondents rate their answers using a 6-point Likert scale. Three of these sub-scales specifically assess core symptoms of eating disorders, while the other eight sub-scales focus on psychological traits commonly associated with eating disorders. This broad array of sub-scales allows for a comprehensive evaluation of both the behavioral and psychological aspects of eating disorders [37]. The scale showed good internal consistency (Cronbach’s alpha ranging from 0.80 to 0.91) and test–retest reliability (ranging from 0.81 to 0.89).

#### 2.2.3. The ORTO-15 Questionnaire

The ORTO-15 is a tool designed to evaluate eating behaviors associated with ON. It includes 15 items, with responses rated on a four-point Likert scale. Lower scores on the questionnaire indicate greater orthorexic symptoms. The questionnaire has been validated using two distinct threshold scores: a score of <35, which ensures greater specificity, and a score of <40, which provides higher sensitivity [38,39]. Later studies on the psychometric properties of ORTO-15 reported a Cronbach’s alpha score of 0.47.

### 2.3. Statistical Analysis

First, we compared gender among the two diagnostic groups using a Chi-square test.

Then, a Student *t*-test was performed in order to compare AdAS Spectrum, EDI-2, and ORTO-15 total scores between the two groups.

Subsequently, we performed another Chi-square analysis to compare how many subjects scored below the threshold of 35 on the ORTO-15, indicating significant orthorexic tendencies, based on their diagnostic group affiliation.

Afterwards, we performed two Pearson’s correlation analyses with the aim of investigating the presence of significant correlations between, respectively, ORTO-15 total scores and EDI-2 domains and total scores, and ORTO-15 total scores and AdAS Spectrum domains and total scores.

Lastly, we performed a linear regression analysis using ORTO-15 total score as the dependent variable and AdAS Spectrum and EDI-2 total scores as independent variables in order to investigate whether the autistic features or pathological eating habits were statistically predictive of greater orthorexic tendencies.

All statistical analyses were performed with SPSS version 26.0.

## 3. Results

BPD and HC participants did not significantly differ in gender (see Table 1).

Results from the Student *t*-test showed how BPD subjects scored significantly higher in the AdAS Spectrum and EDI-2 total scores compared to HCs, indicating a greater presence of autistic traits and altered eating behavior in said group. Similarly, BPD subjects scored significantly lower on the ORTO-15 questionnaire suggesting greater orthorexic tendencies (see Table 2).

Results from a further Chi-square analysis showed that subjects who scored below the ORTO-15 threshold score of 35, indicating significant orthorexic tendencies, were significantly more represented in the BPD group (see Table 3).

Results from the Pearson correlation analysis showed that the ORTO-15 total score negatively correlated with all EDI-2 domains, except for Interpersonal Distrust and Social Insecurity, and the total score. The correlation coefficients ranged from weak to moderate, with the strongest correlations being between ORTO-15 total score and EDI-2 Drive for Thinness domain and total score.

Similarly, the ORTO-15 total score negatively correlated with all AdAS Spectrum domains and total score, with the correlation coefficient being moderate. The strongest correlations emerged between ORTO-15 total score and AdAS Spectrum Childhood and Adolescence, Inflexibility and Adherence to Routine, Restricted Interests and Rumination, and Hyper/Hypo-reactivity to sensory input domains and with its total (see Table 4).

Lastly, results from the linear regression analysis performed using ORTO-15 total score as the dependent variable and AdAS Spectrum and EDI-2 total scores as independent variables highlighted how autistic features were statistically predictive of greater orthorexic tendencies (see Table 5).

## 4. Discussion

The aim of the present study was to investigate orthorexic tendencies and their correlations with autistic traits and altered eating behavior dimensions in a sample of patients with BPD.

As expected, BPD individuals scored significantly higher than HCs in the questionnaire assessing the presence of autistic traits and pathological eating behaviors. These data are not only consistent with the existing literature but also strongly aligned with various findings in the field. A growing body of research has indeed emphasized the overlap between autistic traits and BPD. Specifically, several studies have shown that individuals with BPD often exhibit more pronounced autistic traits [20,40,41]. Furthermore, these studies suggest important similarities between the symptoms and characteristics of BPD and autism spectrum disorder (ASD), including the tendency for individuals with both conditions to form intense, unstable relationships and maintain superficial friendships. Additionally, both disorders share a common pattern where individuals act out or engage in impulsive behaviors instead of verbally expressing their emotions. While these features are typically considered hallmarks of BPD, they are also frequently observed in individuals with ASD [2,20,42,43,44,45,46,47]. Similarly, the well-established connection between BPD and eating disorders is widely recognized [48]. Indeed, many studies have demonstrated the presence of disordered eating behaviors—including binge eating, dieting, skipping meals, and vomiting—in adults with BPD, whether or not they are diagnosed with a co-morbid eating disorder [7]. Moreover, some researchers have pointed to a direct correlation between these two conditions, driven by transdiagnostic factors that overlap in both disorders. These shared factors include socio-cultural influences, common etiological elements, and syndromic components, such as difficulties with emotional regulation, interpersonal challenges, issues with self-concept, and impulsivity [5]. Interestingly, BPD subjects also scored significantly lower than HCs in the ORTO-15 questionnaire, demonstrating a greater prevalence of orthorexic tendencies among this population. Such results were confirmed by the Chi-square test showing that a significantly greater percentage of subjects with BPD scored under the ORTO-15 threshold score of 35 for clinically relevant orthorexic behaviors. Although research on the specific association between BPD and ON is still scant, some promising results are slowly emerging. In particular, a recent study investigating personality profiles in young adults with orthorexic eating behaviors showed that orthorexic people were more likely to manifest personality traits belonging to cluster B [33]. Additionally, ON has not yet been formally classified as a distinct mental disorder. Instead, some researchers have described it as ‘the anorexia of the new millennium’ due to its many clinical similarities with anorexia nervosa (AN), but with a key distinction: ON is generally more socially accepted [27,29]. In this context, the known correlation between BPD and FED could also be applied to ON. Moreover, it is plausible that the presence of elevated autistic traits—which are particularly prominent in BPD and have been suggested to be associated with the development of FEDs [15,49]—may underlie the development of ON [32].

Results from Pearson’s correlation analysis showed that all EDI-2 domains and total scores were significantly negatively correlated with ORTO-15 total score, with the only exception of the Interpersonal Distrust and Social Insecurity domains, suggesting that altered eating behaviors were associated with greater orthorexic tendencies. To date, many studies support the connection between orthorexic tendencies and features of eating disorders, showing a small to moderate correlation between ON and other FEDs [50,51], and highlighting how both conditions may act as reciprocal risk factors [52,53,54]. Additionally, AN and ON share various clinical characteristics, such as strict dietary adherence and malnutrition [55,56], along with psychopathological traits like perfectionism, a desire for thinness, and body dissatisfaction [57,58]. In particular, the Drive for Thinness in orthorexic subjects may be explained by a distorted vision in which weight loss could be seen as proof of success in maintaining a “healthy” diet. Therefore, although ON does not focus directly on thinness as a goal, some people may begin to see the achievement of thinness as a demonstration of the success of their healthy eating and, thus, the desire to be thinner may emerge as part of this obsession with ideal health [29]. While our findings support these observations, they also reveal a significant link between orthorexic tendencies and other psychopathological domains, including Bulimia, Impulsivity, Maturity Fears, and Asceticism.

This is in line with previous studies that highlighted the presence of binging and purging behaviors in ON clinical pictures, as well as the moral connotation attributed to their eating behaviors by ON subjects [59,60]. Interestingly, our findings did not reveal a significant statistical link between orthorexic tendencies and the EDI-2 domains of Interpersonal Distrust and Social Insecurity. One possible explanation can lay in the fact that in Western cultures, behaviors associated with orthorexia are often seen as less harmful or even socially advantageous [61,62]. Recent studies have pointed out the growing influence of healthy eating communities on social media, particularly platforms like Instagram [63]. This, coupled with the fact that ON is not widely regarded as a mental disorder, may reduce social stigma for individuals with ON, leading to more positive social interactions. However, this does not rule out potential issues with social–emotional reciprocity in people with ON. In fact, we observed significant correlations between orthorexic tendencies and various domains of autism, including verbal and non-verbal communication and empathy, as measured by the AdAS Spectrum questionnaire. Indeed, EDI-2 domains focus primarily on an individual’s perception of the quality of their social relationships, while the AdAS Spectrum domains investigate difficulties with mentalizing and communication, regardless of whether these challenges are perceived as problematic. Therefore, individuals with more pronounced ON symptoms may report social difficulties but still consider their social relationship subjectively acceptable, eventually due to the use of social camouflaging strategies, which seem to be associated with the development of FED in previous studies [64] or also to a reduced interest in social relationship or a lack of relationship understanding [15]. However, individuals with ON may exhibit a sense of moral superiority and intolerance toward those who do not share their food-related concerns, which suggests autistic-like deficits in social skills, similar to those seen in AN patients [30].

Moreover, the ORTO-15 total score was also significantly negatively correlated with all remaining AdAS Spectrum domains, as well as with its total, suggesting that greater orthorexic tendencies were associated with higher autistic traits. In particular, the strongest correlations appeared to be with AdAS Spectrum Inflexibility and adherence to routine and Restricted interests and ruminations domains. The link between cognitive inflexibility and ON may be explained in light of some of the core characteristics of ON. Indeed, cognitive inflexibility can make it difficult for individuals with ON to adapt or deviate from their rigid food rules, even when presented with new information or changes in circumstances. For example, if someone with ON is invited to a social event where food choices are outside of their strict guidelines, they may struggle to adjust, leading to anxiety or avoidance. Moreover, subjects with cognitive inflexibility often find it hard to accept exceptions or make exceptions to their rules [35,65,66]. In ON, this manifests in an intense focus on “clean” eating, and any deviation from this rigid food standard may be perceived as a failure or loss of control. Cognitive inflexibility can also manifest in compulsive behaviors, such as spending excessive time planning meals, researching food ingredients, or following strict routines regarding when and how to eat. Lastly, cognitive rigidity has long been associated with other eating disorders such as anorexia nervosa, in which rigidity revolves around extremely restrictive dieting. In this context, given the conceptualization of ON as “the anorexia of the new millennium”, it is possible to hypothesize the presence of similar attitudes of cognitive rigidity also in orthorexic patients [35,65,66]. Similarly, individuals with restricted interests often display a high level of focus on a specific subject, activity, or behavior. In the case of orthorexia, this may manifest as an excessive and rigid preoccupation with eating “healthy” food. Furthermore, ruminations, understood as repetitive and intrusive thoughts, may concern persistent thoughts about food, eating habits, preparation and content of meals, or perceived health risks related to food [29]. This may occur in a similar way to what happens in AN, for which a possible reconceptualization has been proposed as a female phenotypic manifestation of ASD in which the restricted interests concern food and eating [15,21,22].

Interestingly, while a stronger correlation was found between AdAS Spectrum and ORTO-15 total scores than between EDI-2 and ORTO-15 total scores, the regression analysis highlighted AdAS Spectrum total score, but not EDI-2 total scores, as a negative predictor of ORTO-15 score, and therefore as a positive predictor of greater orthorexic symptomatology. Such data align with results from previous studies that reported how greater autistic traits were predictive of greater orthorexic tendencies in various populations [30,35]. It should be noted that while a previous study highlighted that ON seems to be more associated with FED than with obessive–compulsive disorder (OCD) symptoms, our data suggest instead that ON seems to be more associated with the autism spectrum than with FED-related symptoms [67]. These findings align with our expectations, given the established similarities between AN and ON, as well as the increasing research suggesting a strong connection between AN and female presentations of autism spectrum [29,68,69,70]. In this framework, ON may be seen as a manifestation of the same autistic traits that characterize AN. This theory suggests that both disorders share a core set of autistic traits, which are particularly expressed through a focused preoccupation with food and dietary habits [29,71]. The differences in how symptoms are mentally processed between AN and ON, such as restrictive eating patterns based on personal beliefs about the ‘healthiness’ of food or its calorie content, may be influenced by social environmental factors or the severity of the disorder. Thus, ON could potentially be part of the same autistic spectrum phenotype as AN, with both conditions stemming from a common autistic core that manifests in rigid, narrow dietary focus, repetitive behaviors surrounding food, and difficulties with social and emotional interactions [29,35,71].

These results should be seen in light of some limitations. For instance, the cross-sectional design of the study prevents us from making temporal or causal connections between the variables under analysis. Moreover, the self-report nature of the psychometric questionnaires used for the evaluation of the participants may induce an over- or under-estimation of symptoms based on the subjective perception. In addition, the relatively small size of the sample restricts the generalizability of our results, making it difficult to extend these findings to larger, more diverse populations.

## 5. Conclusions

Globally, this study highlights a greater presence of ON symptoms in subjects with BPD, in line with the higher presence of FED in this population. While in our sample ON tendencies seem to be associated with both FED- and autism spectrum-related symptomatology, a higher association was reported with the latter, further stressing the link between autism spectrum and ON symptoms reported in the previous literature [30].

## 6. Recommendations

Mental health professionals should routinely assess for orthorexic behaviors in individuals with BPD, particularly when marked autistic traits or rigid dietary patterns are present. Incorporating orthorexia-related questions into clinical intake procedures may facilitate earlier identification and more effective intervention. Additionally, psychiatric and psychological training programs should enhance clinician awareness of the overlap between neurodevelopmental traits and disordered eating patterns. Improving education on the presentation of orthorexia within the broader autism spectrum may help reduce rates of underdiagnosis and clinical mismanagement.

Future research should prioritize longitudinal studies to clarify the temporal and causal relationships among autistic traits, BPD symptomatology, and the emergence of orthorexic behaviors.

Furthermore, due to the current limitations of orthorexia assessment tools, there is a pressing need to develop and validate more comprehensive and reliable instruments.

## Figures and Tables

**Table 1:** Gender comparison between groups.

		HC mean ± SD	BPD mean ± SD	F	*p*
**Age**		33.62 ± 10.70	30.89 ± 11.61	0.014	0.184
		**n (%)**	**n (%)**	**Chi-square**	***p***
**Gender**	**F**	45 (41.3%)	64 (58.7%)	0.035	0.852
	**M**	7 (43.8%)	9 (56.3%)		

**Table 2:** Comparison of AdAS Spectrum, EDI-2, and ORTo-15 total scores among HCs and BPD patients.

	HC mean ± SD	BPD mean ± SD	F	*p*
**AdAS Spectrum total score**	21.27 ± 17.54	78.38 ± 24.96	14.318	<0.001 *
**EDI-2 total score**	32.48 ± 19.29	78.94 ± 38.87	45.356	<0.001 *
**ORTO-15 total score**	43.46 ± 6.42	36.92 ± 5.28	2.807	<0.001 *

* significant for *p* < 0.05.

**Table 3:** Comparison of ORTO-15 threshold among diagnostic groups.

	No ON	ON	Chi-Square	*p*
**HCs**	48 (92.3%)	4 (7.7%)	9.524	0.002 *
**BPD**	50 (69.4%)	22 (30.6%)		

* significant for *p* < 0.05.

**Table 4:** Pearson’s correlation coefficients between ORTO-15 total score and EDI-2 domains and total scores and between ORTO-15 total score and AdAS Spectrum domains and total scores in the overall sample.

EDI-2	ORTO-15 Total Score	AdAS Spectrum	ORTO-15 Total Score
**Drive for Thinness**	−0.403 **	**Childhood adolescence**	−0.443 **
**Bulimia**	−0.354 **	**Verbal Communication**	−0.346 **
**Body Dissatisfaction**	−0.287 **	**Non-verbal Comm.**	−0.406 **
**Ineffectiveness**	−0.185 *	**Empathy**	−0.382 **
**Perfectionism**	−0.310 **	**Inflex. and adh. routine**	−0.458 **
**Interpersonal Distrust**	−0.019	**Restricted int. and rum.**	−0.478 **
**Interoceptive Awareness**	−0.234 **	**Hyper/Hypo-react.**	−0.433 **
**Maturity Fears**	−0.334 **	**AdAS Spectrum Total score**	−0.467 **
**Asceticism**	−0.345 **		
**Impulsivity**	−0.227 *		
**Social Insecurity**	0.003		
**Total score**	−0.348 **		

* significant for *p* < 0.05; ** significant for *p* < 0.01.

**Table 5:** Linear regression analyses with ORTO-15 total score as a dependent variable and AdAS Spectrum and Edi-2 total scores as independent variables in the overall group.

	B(S.E.)	Beta	t	p	Tolerance	VIF
Constant	44.477 (1.018)		43.685	<0.001 *		
**AdAS Spectrum total score**	−0.081 (0.021)	−0.436	−3.911	<0.001 *	0.518	1.930
**EDI-2 total score**	−0.007 (0.0189)	−0.045	−0.401	0.689	0.518	1.930

R^2^: 0.220; adjusted R^2^: 0.207; * significant for *p* < 0.05.

## Data Availability

All data generated or analyzed during this study are included in the published article.

## Abbreviations

The following abbreviations are used in this manuscript:

BPD	Borderline Personality Disorder
FED	Feeding and Eating Disorders
ASD	Autism Spectrum Disorder
ON	Orthorexia Nervosa
HC	Healthy Controls
AdAS	Adult Autism Subthreshold
EDI	Eating Disorder Inventory
AN	Anorexia Nervosa
